# Patterns of Major Oral and Maxillofacial Surgery Cases in Two Ethiopian Medical Centers: A Three-Year Retrospective Study

**DOI:** 10.4314/ejhs.v34i2.4

**Published:** 2024-03

**Authors:** Surafel Adula, Demerew Dejene, Girma Uma, Gelana Garoma

**Affiliations:** 1 Department of Oral and Maxillofacial surgery, College of Health Sciences, Addis Ababa University, Addis Ababa, Ethiopia

**Keywords:** Oral and Maxillofacial, surgery, patterns, trends

## Abstract

**Background:**

Oral and Maxillofacial region is an area that is prone to multitude of disease conditions of developmental nature or acquired which require surgical intervention on many occasions. The aim of this study was to assess the pattern, indications, and types of major oral and maxillofacial surgical procedures carried out at Addis Ababa university Maxillofacial Surgery affiliate hospitals.

**Methods:**

A retrospective study was conducted on all patients who had undergone oral and maxillofacial surgeries under general anesthesia at Yekatit 12 Hospital Medical College and Saint Peter Specialized Hospital in a period of 3 years from January 2017 to December 2019. Descriptive statistics such as biodata, indication and type of surgery were recorded and analyzed using the Epi info version 7 software.

**Results:**

A total of 689 patients with oral and maxillofacial conditions were managed during the study period. The male to female ratio was 2.48:1, and the mean age of patients was 29.05 years with standard deviation of ±15.5. The predominant indications for surgery were traumatic conditions (50.71%, n=354), followed by neoplastic conditions (18.7%, n=129)). Open reduction and internal fixation were the most common (43.7%, n=313) surgical procedure performed.

**Conclusions:**

The field of oral and maxillofacial surgery in our climes is a relatively new one. However, it is evolving, and its relevance is growing. With the observed relatively high frequency of traumatic conditions, especially among younger populations, efforts geared at prevention and adequate preparedness for proper surgical management of such patients should be commenced.

## Introduction

Oral and maxillofacial (OMF) region is an area that is prone to multitude of disease conditions which may be of developmental nature (like vascular anomalies and Orofacial clefts) or acquired nature (such as neoplasms, trauma and infections) which require surgical intervention in many occasions. Thus, the scope of OMF surgery includes the diagnosis and treatment of diseases affecting the mouth, jaws, face, and neck (1).

Diseases requiring surgical intervention have been estimated to account for 11% of the global burden of disease (2). Despite the magnitude of these estimates, surgery remains a neglected aspect of health systems development in low- and middle-income countries in which inadequate equipment and workforce remain a major challenge in surgical capacity building (3,4). Ethiopia, as a developing country and maxillofacial surgery as recently commenced specialty training in the country (the first cohort of OMF surgeons graduated from Jimma University in 2016), it is not an exception to this challenge (5).

In modern healthcare systems, there is a growing emphasis on monitoring the outcomes of health intervention in general and surgical procedures in particular. As healthcare evolves and the demands on the available facilities increase, the need for evaluating existing health systems in order to improve their efficiency becomes more obvious (6,7).

To achieve this, there is often a need to assess the demands being placed on the system, the challenges being experienced, and the successes being accomplished. A retrospective review of surgical services provided is one of the ways of ensuring optimal or improved service delivery (8).

Although few studies examined the pattern of distribution of specific member of the spectrum such as orofacial neoplasm and odontogenic tumor (9,10), trauma (11), oral cancer (12) and temporomandibular joint (13), there is a dearth of information on the total spectrum of OMF surgical procedures in Ethiopia. This study was therefore undertaken with an aim of reporting on the indications, pattern and types of major OMF surgical procedures carried out in Yekatit 12 Hospital Medical College and St. Peters Specialized Hospital, which are affiliates of Addis Ababa University Tikur Anbessa Specialized Hospital, OMFS Department.

## Materials and Methods

**Study area, design and period**: The study was conducted at Yekatit 12 Hospital Medical College and St. Peter Specialized Hospital, which are an affiliate of Tikur Anbessa Specialized Hospital. These affiliate hospitals are in the city of Addis Ababa providing emergency and elective maxillofacial care. Enrollment of maxillofacial surgery residents is a recent development of the Tikur Anbessa Specialized Hospital, which marked the initiation of specialty training in the College of Health Sciences, Addis Ababa University, for the first time. A retrospective cross-sectional descriptive design was used. The study was undertaken from November 2019 to August 2020.

**Study population**: All patients who underwent maxillofacial surgery at Yekatit 12 Hospital Medical College and St. Peters specialized Hospital during the study period whose document fulfilled the inclusion criteria. A total of 704 patients who were operated on were included as study participants.

**Sample size and sampling technique**: No sample size was determined as the all-source population was included as the study subjects provided that the documents were complete for the variables of interest.

**Inclusion and exclusion criteria**: All patients who underwent major maxillofacial surgery at specified area and period were included. Out of total of 15 operated on patients whose documents had missing or incomplete of any the information- age, sex, indications of surgery (diagnosis) or types of performed surgery were excluded.

**Data collection and analysis**: The data was collected by five personnel: principal investigator and four dental interns using predesigned and structured checklist (format) on which required variables were filled from the patient registry logbooks. The checklist for data collection consists of age, sex, diagnosis type, and procedure performed. Completeness of data was checked by the principal investigator, and orientation was given to other colleagues involved in the study. After data collection was finished, cleaning and checking of the content was done. Further data cleaning was performed to check for missed values and any data inconsistency before data analysis. Finally, data was entered into EPI INFO version 7.2 software, and analysis of different variables was done.

## Operational Definitions

**Types of surgery**: It is a kind of surgical procedure performed for patients who presented with maxillofacial diseases and conditions.

**Patterns of surgery**: It is the frequency and repetitiveness of the surgical cases and surgical procedures performed for the cases.

**Trends of surgery**: It is the frequency distribution of maxillofacial surgical procedures performed over a defined time period, usually annually, of similar duration at different times.

**Ethical considerations**: Ethical clearance was obtained from Addis Ababa Public Health Research and Emergency Management Directorate for Yekatit 12 Hospital Medical College and Ethical Review Committee Office of St. Peter Specialized Hospital.

## Results

**Figure 1:**
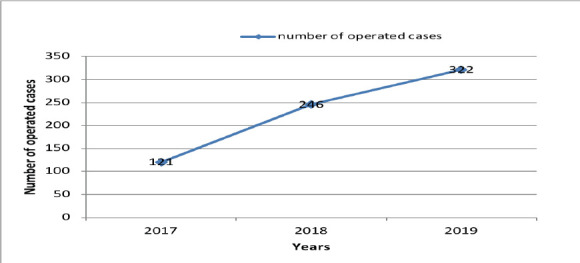
Number of cases operated per year in Two Ethiopian Medical centers, Addis Ababa, Ethiopia, 2020

In this study, data of 689 patients who had undergone surgical procedure under general anesthesia (GA) was analyzed. Males (71%, n=491) were significantly more than females giving a male to female ratio of 2.48:1. The age range of the patients was from 04 months to 84 years with a mean age of 29.05 years and standard deviation of ±15.5. There was a significant difference on indication of surgery between male and female (70.99% vs. 29.04%). There is a statistically significant association between age groups and indications of surgery except for the groups from 51-60, 61-70 and above 81. The most common age groups managed were 21-30 years (35.85%, n=247) and 31-40 years (19.01%, n=131) (Table 1). On average, about 230 cases were operated on per year, with the number of OMF surgeries performed per year increasing from 121 cases in year 2017 to 322 cases in year 2019 (Figure 1).

**Table 1:** Demographic characteristics of patients operated in Two Ethiopian Medical centers, Addis Ababa, Ethiopia, 2020

Variables	Category	Frequency (%)
Gender	Female	198(29%)
	Male	491(71%)
Age (years)	0 – 10	53(7.7%)
	11 – 20	111(16.11%)
	21 – 30	247(35.85%)
	31 – 40	131(19.01%)
	41 – 50	64(9.28%)
	51 – 60	31(4.5%)
	61 – 70	40(5.81%)
	71 – 80	10(1.45%)
	81 – 90	2(0.29%)
	Mean ± SD	29.05 ± 15.5

**Figure 2:**
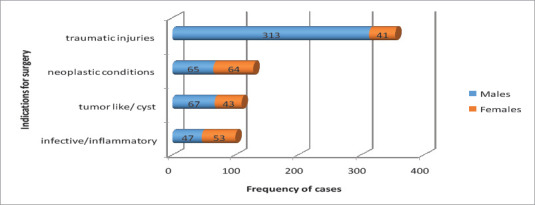
Distribution of surgical indications to gender in Two Ethiopian Medical centers, Addis Ababa, Ethiopia, 2020

More than half of the OMF cases managed surgically were traumatic conditions (51.08%, n=354), followed by benign and malignant neoplastic conditions (18.61%, n=129). The common conditions that were surgically managed in males were trauma (63.61%, n=313), followed by tumor like/cystic conditions (13.61%, n=67), while females were frequently operated for neoplastic conditions (31.84%, n=64) and inflammatory/infective conditions (26.36%, n=53). Furthermore, trauma was the fewest condition (20.39%, n=41) managed surgically in females in contrary to that in males (Figure 2). Traumatic injuries were the most common indication for the majority of age groups with the exception of age groups above 61 years, who were frequently operated on for benign and malignant neoplastic conditions (Table 2).

**Table 2:** Distribution of surgical indications to age in Two Ethiopian Medical centers, Addis Ababa, Ethiopia, 2020

Age group	Variables	Indications of surgery			Total	P-value	

	Injuries	Neoplasia	Tumor like/cystic	Infective/inflammatory			
0-10	20	9	7	17	53		0.03
11-20	42	31	24	14	111		0.001
21-30	164	27	34	22	247		0.000
31-40	79	19	22	12	132		0.000
41-50	29	12	13	10	64		0.002
51-60	9	8	6	9	32		0.86
61-70	11	15	4	12	42		0.102
71-80	0	6	0	4	10		0.012
81-90	0	2	0	0	2		0.11
Total	354	129	110	100	693		

During the study period, more than 50 different types of OMF conditions were managed under GA. The most frequently operated on condition was mandibular fracture (32.2%, n=223), followed by combined zygomatico maxillary complex and panfacial fracture (12.5%), space infections and osteomyelitis (5.4%), ameloblastoma (4.3%), and odontogenic keratocysts (3.9%) (Table 3).

**Table 3:** Oral and Maxillofacial conditions that were commonly managed surgically in Two Ethiopian Medical centers, Addis Ababa, Ethiopia, 2020

Category	Cases	Frequency (%)
Trauma	Mandibular fracture	223 (32.2%)
	ZMC	47 (8%)
	Panafacial fracture	40 (5.7%
	STI	15 (2.1%)
	Midface fracture	13 (1.8%)
Neoplasia	Ameloblastoma	30 (4.3%)
	Pleomorphic adenoma	22 (3.1%)
	Squamous cell carcinoma	14 (2.0%)
	Fibrous dysplasia	11 (1.5%)
Cyst	Okc	27 (3.8%)
	Dentigerous cyst	24 (3.4%)
	Radicular cyst	17 (2.45%)
	Nasopalatine duct cyst	10 (1.4%)
Others	Infection & osteomyelitis	38 (5.4%
	TMJ ankylosis	17(2.4%)
	Sailolithiasis	13 (1.8%)

ZMC - Zygomatic complex, STI - soft tissue injury, OKC - odontogenic Keratocsyt, TMJ -Temporomandibular joint disorder

**N.B:** the conditions presented are those with frequency of above 10

In the total of 689 patients/cases with primary diagnosis, 716 surgical procedures were performed. There were cases that had two surgical procedures at the same time. Four patients had complications after first surgery and operated on again making the indication of surgery 693. Out of 716 surgical procedures performed, open reduction and internal fixation (ORIF) was the most common (43.7%, n=313), followed by cystectomy (11.45%, n=82) and tumor excision (9.1%, n=65). Procedures that involved jawbone resection (mandibulectomy and maxillectomy) were performed on 55 patients. Surgical (total or partial) excision of different salivary glands and incision and drainage were performed in (6.3%, n=45) and (3.0%, n=22) cases, respectively. Sequestrectomy (2.2%, n=16), surgical shaving (1.67%, n=12), and neck dissection (1.3%, n=9) were among the procedures performed with lowest frequencies (Table 4).

**Table 4:** Frequently performed Oral and Maxillofacial surgical procedures in Two Ethiopian Medical centers, Addis Ababa, Ethiopia, 2020

Type of surgical procedure	Frequency (%)
ORIF	313 (43.7%)
Cystectomy	82 (11.5%)
Tumor excision	65 (9.0%)
Sialadenectomy	45 (6.3%)
Mandibulectomy	37 (5.2%)
Debridement & repair	31 (4.3%)
Closed reduction	23 (3.2%)
Incision & drainage	22 (3.0%)
Maxillectomy	18 (2.5%)
Arthroplasty	17 (2.4%)
Sequestrectomy	16 (2.2%)
Surgical shaving	12 (1.7%)
Neck dissection	9 (1.3%)
others	26 (3.6%)

## Discussion

Oral and maxillofacial region is an area that is prone to a multitude of disease conditions which may be of developmental or acquired, including vascular lesion, neoplasms, trauma, cyst and infections. These conditions require surgical intervention on many occasions. This study was undertaken to analyze the indications and pattern of OMF surgeries in our institute, which can be considered as the beginning of efforts to put forward in characterization of the most prevailing maxillofacial diseases and ultimately its detection, treatment and possibly prevention.

In this study, 689 patients with maxillofacial conditions were operated on with 230 surgeries performed per annum in average. The number of operated on cases steadily increased from 121 in the first year to more than two-fold (n=246) in the second year and almost three folds in the third year (n=322). The obvious increase in the number of cases operated per annum in our institutes can be explained by recruitment of more trained OMF surgeons in both Tikur Anbessa Specialized Hospital and two affiliate hospitals (from two surgeons in 2017 to four at 2020) and initiation of the OMF surgery service at the second affiliate hospital (St. Peter Specialized Hospital) in the second quarter of the first study year. Other reasons include increasing awareness of maxillofacial surgery specialty and the services offered among the populace and the medical community.

The results of the current study revealed that there were more male patients than their female counterparts. The result differed from what was reported by Moshy et al (14). Similar results were reported in other studies (15, 16). The ratio of male to female differed in all the studies, and this can be attributed to differences in study design, inclusion criteria and socioeconomic status.

The age range of the patients is similar to reports in the literature, with most of the patients being in the 3rd decade of life. However, some other authors reported varying modal decades in their studies. The study conducted in Nigeria reported the 4th decades of life as the modal decades (17). Rehmann et al. reported 1st decades of life as the modal decades in their studies (18). Notably, they included minor surgical procedures, including routine dental extraction in their study. This may have skewed the modal age group recorded.

Maxillofacial trauma was the principal indication for surgery in this study (50.7%, n=354), similar to the findings of another study (19). The relatively higher proportion of maxillofacial injuries may be because both hospitals at which this study was conducted are found in an urban area, where population is exposed to the hazards of high-speed travelling. Furthermore, most of the cases of maxillofacial fractures are observed in the younger age groups who are active and likely to involve in interpersonal violence and various risky job (20,21).

Mandibular fractures were more commonly encountered than any other maxillofacial fractures (63%, n=223). This finding is comparable to other reports in the literature (22). However, it is at variance with some other studies where nasal bone fractures were reported to be the most frequently encountered maxillofacial fracture (23). Also, Gassner et al. (24) reported maxillary fractures to be the most frequently diagnosed injury site in their study, which was only (3.67%, n=13) of the total injuries in this study. Mandibular fracture is believed to occur frequently because of its prominence (25,26).

Neoplastic conditions, most of which were benign (72.8%, n=94), were the second most frequent indication for surgery in this study. This is at variance with some studies in the literature, where it were in the majority (27). The most commonly observed benign tumors were ameloblastoma (32%, n=30). Ameloblastomas are among the most common benign tumors and its higher prevalence can be explained by its heterogeneous epithelial origin (28). Conversely, Saleh et al., in a study of biopsied oral and maxillofacial lesions reported that the most common benign tumor was keratocystic odontogenic tumor (29). The cases of malignant neoplasms were also treated surgically, though not frequent (5.1%, n=35). These cases were just a portion of all malignant neoplastic conditions that received surgery, where patients with advanced malignancies causing severe morbidity, deformity and at times metastasis to other organs and as such rendered inoperable, receive palliative treatment because of their late presence. In agreement with the findings from other studies (15,30), the common malignant condition that was managed surgically was squamous cell carcinoma (40%, n=14) in our center.

Studies on oral and maxillofacial disease have included limited data on the prevalence of odontogenic cysts in all populations (31). In this study, cystic and tumor-like conditions contributed a significant proportion of indications for surgery (16%, n=110) with commonest entities being Odontogenic keratocyst (24.55%, n=27), dentigerous cyst (21.8%) and radicular cyst (15.4%) in decreasing order. This is not consistent with findings in literatures that found out radicular cyst and dentigerous cyst as the leading cysts among others (32). This could be explained by the fact that only few proportion of radicular cyst, which are not comfortable for patients and the surgeon for removal under local anesthesia, are operated under GA in our center.

The prevalence of space infection and osteomyelitis is significantly scanty (5.48%, n=38) in this study, since most of the infective cases were operated in minor surgery under local anesthesia and were not admitted to the in-patient department.

The findings of this study elucidate that the most frequently performed surgical procedure was ORIF, which demands direct exposure of the fracture site followed by alignment of the bone segments and fixation (33). The cases that were managed by ORIF included all those with unfavourable fracture of mandible and midfacial bone fractures. In the current study, most cases of ORIF were performed using intra-osseous wires (83.07%) due to limited availability of metal implants (plates and screws). Closed reduction was only done in 23(3.2%) of patients with maxillofacial trauma. There are some procedures that can be performed under GA, but still are regarded as closed reduction like, nasal bone reduction, zygomatic arch elevation and inter maxillary fixation. This is in agreement with finding from another study (14), but not in line with the study conducted in Nigeria (34). This might be due to indication, fracture type, age of patients, set up and surgeon's experiences.

Mandibulectomy (n=37) contributed a significant proportion of the surgical procedures in this study. Ameloblastomas, being the most common benign tumor, was managed by performing mandibulectomy, since its cure is undoubtedly surgically. There are various proposed treatment options for ameloblastomas, ranging from conventional to radical methods of therapy. The traditional techniques involve curettage, enucleation and cryosurgery, while the extreme techniques are marginal, segmental and composite resections (35). In our centers, the majority of ameloblastomas were treated radically.

In consonance with other similar studies (36), the relatively fewer numbers of salivary gland surgeries (6.28%, n=45) done may be related to the fact that the specialty of oral and maxillofacial surgery is comparatively new in our country and that a large percentage of the salivary gland surgeries are referred to other surgical specialties for treatment. With the continued development of the maxillofacial surgery specialty in our locality and the attendant increase in awareness, it is believed that more salivary gland surgeries will be done at the OMFS Department.

Even though no single standard treatment protocol for TMJ ankylosis has been reported until now due to high reankylosis rate, a number of treatments for this condition have been described in the literature, including simple arthroplasty, interposition arthroplasty and reconstruction of the joint using acrylic, titanium, or autogenous material prostheses in end stage diseases (37). In the institutes where this study was carried out, the most preferred method was interpositional arthroplasty (64.71%) in agreement with reports of other studies (38).

The frequency of performing hard and soft tissue reconstructive surgeries is fairly low in our institute, whereby some of the patients who had mandibulectomy procedure had reconstruction of the mandible with reconstruction plates. Microvascular surgery offers considerable advantages over the traditional methods of maxillofacial reconstruction (39). However, it was not utilized in this study due to a number of challenges such as resource limitations and inadequate staff.

In conclusion, the field of Oral and Maxillofacial surgery in our climes is a relatively new one. However, it is evolving, and its relevance is growing. There has been an annual increase in the number of OMF surgical procedures performed in our institutes. With the observed relatively high frequency of traumatic conditions, especially among younger populations, efforts geared at prevention and adequate preparedness for proper surgical management of such patients with regard to armamentarium, emergency theatre arrangement, professional and support staff training should be commenced. The department should exert all the possible efforts to avail necessary equipment, train staff and launch advanced procedures like microvascular surgeries.

The limitations of this study were that it was a hospital-based study, and its design was cross-sectional. As such, it is difficult to generalize the findings to the entire population; it represents a specific study group limited to only those undergoing OMF surgery procedures under GA. Secondly, there were some operation lists that had been incomplete and missing. Overall, the present study cast the light on the spectrum of OMF surgeries that were performed in our institutes. The results of this study will aid the relevant authorities to make appropriate evidence-based plans and decisions.
